# Social needs screening in primary care: A tool in the fight for health equity?

**DOI:** 10.1016/j.puhip.2024.100466

**Published:** 2024-01-24

**Authors:** H. Painter, E. Parry, L. McCann, A. Dehn Lunn, J. Ford

**Affiliations:** a Wolfson Institute for Population Health, Queen Mary University of London, UK; b Primary Care Centre Versus Arthritis, School of Medicine, Keele University, Staffordshire, UK

**Keywords:** Social needs screening, Health inequality, Social determinants of health, Primary care

## Abstract

Progress on addressing health inequalities is slow and in many places around the world the gap between the privileged and the disadvantaged is widening. This is driven largely by an unfair and unequal distribution of the social determinants of health. While upstream policy and agenda commitment is needed to improve social determinants of health at a population level, healthcare also has a role. Currently social information is sporadically collected and used in healthcare. Improving our understanding of social problems is crucial in targeting services and to reduce the overreliance on area-level measures of deprivation. This has the potential to improve patient care as well as more accurately capture socio-economic disadvantage. Here we argue that there is a role for primary care in screening for social needs to help address inequalities.

Social needs screening, more commonly used in North America than Europe, aims to systematically collect social information in health and care settings. Healthcare professionals ask patients about social issues including employment, finances, housing, education and social isolation and this information is used to prompt referral to community services to address any need identified.

Social needs screening has potential to address negative impacts of social determinants of health at an individual and population level. Providing a reliable measure of social need, screening gives healthcare professionals an opportunity to tailor and improve quality of care for patients and offer individualised support. It has been shown to improve individual social and health outcomes and positively impact healthcare utilisation. At a population level, social needs screening can improve the data on social determinants of health and therefore support policy makers and service delivery leaders to target resources and services more effectively to the communities most in need. Implementing social needs screening must take account of local healthcare service capacity and available community resources but where sustainable, effective programmes can be introduced, the potential benefits are manifold.

While primary care alone cannot solve the root causes of health inequalities, we argue it could be a powerful actor in the fight for health equity.

## 1 Health inequalities and social determinants of health

Health inequalities are avoidable, unfair and systematic differences in health between different groups of people [1]. They are experienced between and within populations and are driven by inequalities in social determinants of health (SDoH). SDoH are the conditions in which people are born, grow, work, live and age, and the broader societal, economic and political forces and systems that shape this [2]. These factors are fundamental in shaping health [3,4] and are arguably more influential on health outcomes than healthcare itself [5].

Health inequalities go far beyond the health sector – they are impacted by economics, domestic and foreign policies, and social values. Changes in national and global economics have profound impacts on the health of populations. Low socioeconomic status is a known cause of premature mortality worldwide [3], and is thought to contribute to 1 in 3 premature deaths in the UK [6]. A Scottish study published this year, modelling how wage inflation and price inflation may impact household income and spending power, predicted that those in the most deprived areas will suffer the most. The model estimated that premature mortality rates could rise by up to 23 % in the most deprived areas and decrease life expectancy by 2.1 %. Even with government financial support such as the energy price guarantee and cost of living support package, premature mortality rates were predicted to rise by 8 % and life expectancy predicted to fall by 0.9 % [7].

While global crises and domestic politics rumble on, addressing the negative impacts of an unequal distribution of SDoH from a healthcare perspective can feel overwhelming and hopeless. In this article we argue that healthcare has a valuable role in improving our understanding of inequalities in SDoH and guiding action. One approach to this is social needs screening.

## 2 Understanding the scale of the problem

There is a paucity of information available to healthcare professionals and decision makers on people's SDoH and their unequal distribution within society. Historically individual information about SDoH like finances, housing difficulty, education, employment and social isolation are not recorded systematically in healthcare settings. If they are recorded it tends to be ad-hoc, or area-based deprivation scores are used and applied inappropriately to individuals. The ecological fallacy states that if a particular group of people living in an area are highlighted as having poorer health outcomes, it is an error to conclude that a randomly selected individual will necessarily have poorer health outcomes than that for the overall population. As such, area-level measures of deprivation, such as the Index of Multiple Deprivation, are less reliable in identifying individual disadvantage and can underestimate levels of individual poverty [8,9]. Nevertheless, neighbourhood factors and measures remain important and previous research has shown the important and independent effect of area-based socio-economic position compared to individual socio-economic position [10]. Similarly, the findings on the relative benefits of individual versus area level data to predict health outcomes often vary depending on the SDoH and outcome being measured [[11], [12], [13]].

Collecting self-reported information about individual social needs has been found to be more effective than objective measures, such as income or job role, in predicting health outcomes [14]. If collected systematically, individual, self-reported social needs information could deepen our understanding of the distribution of SDoH, their impact on health outcomes and assist in interventions to reduce inequalities at a population-level. Without reliable data on the scale and nature of the root causes of health inequity, the planning of resources, services and policies to address them is likely to be poorly targeted and ineffective.

## 3 The potential for primary care

Primary care is often the first point of contact for people seeking help with problems much broader than medical care, particularly as practitioners often build trust over time with the people and the communities they serve. Even if not the initial presenting concern, social issues often present as stress or contributors to other medical problems. A pre-pandemic survey of General Practitioners in the UK found that approximately 1 in 5 appointments were for non-medical issues, relating predominantly to social needs, costing £400 million per year [15]. This is likely to be much higher in more deprived areas with higher proportions of disadvantaged groups [16] and reflects an opportunity and a need to address the SDoH, and their unequal distribution, in primary care.

There are many examples of primary care organisations, often in disadvantaged communities, that have developed innovative ways of providing enhanced support for patients with complex social needs [17], with social prescribing being a well-known example. Despite anecdotal examples of benefit of these interventions, the evidence is limited [[18], [19], [20]], in part due to a lack of comprehensive data on SDoH to support rigorous evaluation.

There is momentum in primary care towards greater integration between services to improve health inequalities and access to care, highlighted in the UK by the recent Fuller Stocktake [17]. This emphasis on closer relationships between health and social care and data driven approaches underpins the importance of interventions such as social needs screening for improved proactive, integrated care.

## 4 Social needs screening

One way to collect SDoH data across primary, secondary and social care is social needs screening. This involves people or their caregivers being invited to answer questions about a range of social needs such as financial difficulty, housing and education. This information can then be used to identify those who may benefit from social support, tailor clinical decision making and prompt referral or signposting.

Social care is more familiar with the concept of social needs screening and programmes such as ‘Supporting Families’, a UK government funded social care scheme, uses an example of screening to assess the needs of families referred to them and address specific concerns [21]. Going beyond screening those who seek help, some UK local governments are using a ‘Low Income Family Tracker’ [22]. This is an innovative approach to using data on debt and benefits to proactively identify those in need, with significantly greater improvements in household debts seen compared to a control group [23].

In contrast, formalised social needs screening has not been prominent in the health sector but is becoming more common, particularly in North America; since 2014 the Institute of Medicine has had recommendations for collecting social needs information [24]. Subsequently, tools such as the Protocol for Responding to & Assessing Patients' Assets, Risks & Experiences (PRAPARE), the Well Child Care, Evaluation, Community Resources, Advocacy, Referral, Education tool (WE CARE), and the Accountable Health Communities screening tool (AHC) are widely used [[25], [26], [27]]. Screening questions covered in these tools cover a range of domains including economic stability, education, food, neighbourhood and environment and social and community.

Social needs screening in healthcare is generally effective at identifying those requiring additional social support [28], increases referral rates to interventions or community-based services, and leads to resolution of self-reported problems [18,29]. It can also improve quality of consultations by ensuring a personalised care approach, prompting longer appointment times where needed, encouraging a focus on preventative care and enabling clinicians to be mindful of potential barriers to healthcare such as prescription costs [30].

Although the evidence is more mixed when evaluating the impact of social needs screening on health outcomes, the trend is towards studies showing benefit, including demonstrated improvements in a wide variety of areas such as self-reported child health, smoking cessation, depression, blood pressure and lipid control [29]. There are fewer studies looking at system-wide outcomes but there is evidence that screening can improve adherence to treatment regimens, increase immunisation rates, reduce A&E attendance and reduce hospital readmissions [18,29].

## 5 Challenges with social needs screening for primary care

Despite its clear potential and an urgent unmet need for improvements in SDoH, social needs screening has not been widely implemented outside of North America. Time constraints, physician discomfort or lack of expertise are frequently cited reasons for not screening [30,31]. Health systems worldwide are often overwhelmed with demand for appointments, lack of resources and increasing complexity meaning policy makers, planners and clinicians can be distracted away from preventative care, even though this is likely to save time and resources in the long term.

For a social needs screening programme to be successful in primary carel, integration into existing workflows is important. Many would advocate for a multi-disciplinary and multi-sector approach to avoid over reliance on clinicians. This could be through administrative staff, social prescribers, or key workers collecting information, or using innovative tools such as self-administered online or text forms [[31], [32], [33]]. In many settings community health and social care services already collect SDoH data as part of managing, for example financial welfare programmes, child protection services and obstetric care. Ultimately, linking and consolidating data between these systems will be fundamental in collating a comprehensive dataset and though challenging will be of great long-term value.

Linked to this is a challenge in the substantial heterogeneity of current data collection; in North America there are numerous screening tools in use which collect on a range of different SDoH from different populations, and the majority are unvalidated [34,35]. The potential population level benefits to social needs screening will be best achieved if there is consistency in collection with a validated, context-appropriate tool. Recording of ethnicity in primary care records has been successful in the UK predominantly due to financial incentives, effective integration into existing workflows, and effective leadership from administrative teams [36]. A crucial difference is that ethnicity is recorded once for each patient, whereas social needs information will need updating regularly. This poses further questions about how often this is done to avoid screening becoming a tick box exercise. Creating and maintaining motivation could be a potential barrier and should be acknowledged and mitigated through design of well-funded, integrated and multi-disciplinary programmes. Context-specific evidence of the benefit of social needs screening through individual staff and patient experiences of using it, as well as high-quality research, is also likely to be instrumental in driving motivation of providers.

There have been concerns that people would find questions from their health providers about their finances, housing and other personal areas of life intrusive, although generally studies have found screening to be acceptable to patients [37]. We must further consider if people are identified as having a social need, would they want support from their healthcare provider to resolve that? Although one small study found that only 3 % of patients who screened positive for social needs wanted onward help or referral [30] another based in accident and emergency found that 75 % of patients requested help [38]. This variation may be context or provider dependent and emphasises the importance of local programme evaluation.

The variation in patients’ asking for help after identifying a social need may also reflect a lack of faith in being offered a viable solution. Prior to introducing any screening test we must consider the fundamental principles of screening to ensure the benefits outweigh the risks [39]. Without effective and well-resourced social care and community organisations to address the issues highlighted by screening should we be asking people these questions at all? For example, a referral to a social prescriber will only help to alleviate housing difficulty if there is provision within the local authority or charity sector to provide a solution. If this is not the case social needs screening could give patients false hope and fruitlessly overburden social care services, causing more harm than good. Furthermore, we should consider the potential moral injury to practitioners whereby stress can build-up if healthcare providers identify a problem but are restricted in their ability to help. The catch-22 may be that we need to first collect better data on inequalities in SDoH and the impact of this to leverage the agendas of health and social care leaders and policy makers and advocate for the much-needed expansion of resources in the community to support social needs.

## 6 The fantasy paradigm: healthcare intervention will only take us so far

The 2008 WHO report ‘Closing the Gap’ advocated three approaches to addressing the negative impacts of unequal SDoH and achieving health equity: improve daily living conditions; tackle inequitable distribution of power, money and resources; and measure and understand SDoH and assess the impact of action [3]. Social needs screening could contribute towards addressing the third of these but a sustained upstream policy commitment to addressing the first two approaches is required to mobilise progress in addressing inequalities in SDoH [40].

The ‘fantasy paradigm’ in health inequalities refers to the theory that downstream interventions cannot have large-scale meaningful impact without upstream, long-term policy changes that address SDoH at the root cause, and to perpetuate ideas to the contrary is supporting a convenient ‘fantasy’ [40]. Even more, there is evidence that some downstream public health interventions aimed at reducing inequalities can actually exacerbate them [41], therefore careful consideration and evaluation of health system interventions is essential to ensure equity-driven improvement.

While existing structures and workflows in primary care could provide an opportunity to facilitate improvements in SDoH and health inequalities through examples such as social needs screening, it is important to reinforce that multi-sector collaboration and wider geopolitical action are needed for meaningful change. In the example of social needs screening, primary care teams would need to work closely with secondary care, public or social services and the voluntary, community and social enterprise sector. Without capacity, collaboration and motivation in these allied sectors, primary care alone will become overwhelmed by the workload and unable to provide interventions for social needs. 10.13039/100014337Furthermore, top-level policy support and systemwide leadership is needed to adequately resource and advocate for new programmes, ensure consistency in data recording, facilitate sharing of information between sectors and support rigorous evaluation of impact.

Beyond the potential of social needs screening and other social and healthcare interventions we must not lose sight of the fact that sustained, meaningful, equitable improvements in health inequalities requires policies that address systemic social power imbalances and poverty. Every sector has a role in our pursuit of a fairer and more equitable society.

## 7 Conclusion

The impact of health inequalities and their origins in the unequal distribution of the SDoH has long been known, however progress towards addressing these inequalities remains slow or is even going backwards [3,42]. While upstream policy action directed at addressing root causes of inequalities in SDoH is what is ultimately needed, there is a role for the healthcare and research community in ensuring that practice promotes health equity. This should include improving the understanding of SDoH, and leveraging this knowledge to advocate for more, better targeted resources and ultimately policy change. In addition, we need to expand interventions that are proven to reduce inequality. Social needs screening has the potential to facilitate these goals this if well designed, integrated into existing workflows and with multi-sector collaboration. Future research should be directed towards context-specific evaluation of social needs screening in primary care to determine firstly whether it is feasible and secondly whether it can have a positive impact on the inequities caused by SDoH.

Primary care alone cannot solve the wider issues in our societies, or the root causes of health inequalities, but has potential to be a valuable tool in the fight for health equity.

## Declaration of competing interest

The authors declare that they have no known competing financial interests or personal relationships that could have appeared to influence the work reported in this paper.
